# Supplementary material for: Child anemia in Cambodia: A descriptive analysis of temporal and geospatial trends and logistic regression-based examination of factors associated with anemia in children
Source: PLOS Glob Public Health. 2023 Sep 15;3(9):e0002082. doi: 10.1371/journal.pgph.0002082 (PMC10503718; doi:10.1371/journal.pgph.0002082)
Supplement: S1 Table — (DOCX) [file pgph.0002082.s001.docx]

**S1 Table.** **Results of checking multicollinearity using Variance Inflation Factor**

| **Variable** | **VIF** |
| --- | --- |
| Wealth quintile | 2.13 |
| Type of toilet facility | 1.96 |
| Children underweight | 1.63 |
| Birth order | 1.56 |
| Mother age | 1.50 |
| Children stunted | 1.45 |
| Mother education | 1.36 |
| Region | 1.24 |
| Children wasted | 1.2 |
| Place of residence | 1.18 |
| Recent diarrhea | 1.11 |
| Child age | 1.10 |
| Recent fever | 1.09 |
| Source of drinking water | 1.09 |
| Maternal anemia | 1.02 |
| Sex of child | 1.00 |
