# Supplementary material for: Child anemia in Cambodia: A descriptive analysis of temporal and geospatial trends and logistic regression-based examination of factors associated with anemia in children
Source: PLOS Glob Public Health. 2023 Sep 15;3(9):e0002082. doi: 10.1371/journal.pgph.0002082 (PMC10503718; doi:10.1371/journal.pgph.0002082)
Supplement: Attachment — Submitted filename: Manuscript Number PGPH-D-23-00986_Comments.docx [file pgph.0002082.s003.docx]

**"Child Anemia in Cambodia: A Descriptive Analysis of Temporal and Geospatial Trends and Logistic Regression-Based Examination of Factors Associated with Anemia in Children Age 6-59 Months"**

Review comments

**Line 69**

Which period? Please specify the period and reference

**Line 136-140**

Can SINGLE, WIDOWED and SEPARATED women be considered married?

In reality their social status is "NOT MARRIED" as they are not married.

Same comments with OCCUPATION

It would be good to make the following groups:

1. NOT WORKING (NO INCOME)

2. WORKING (others who work and earn money for family needs, so they have INCOME)

**Line 145-146**

Place of birth should be “HOSPITAL" and "HOME"

Being born in a public or private hospital is the same government objective (birth assisted by qualified personnel).

Indicators monitor are “Delivery assisted by qualified health worker” and “Delivery unassisted by qualified worker”

**Line 148-150**

It would be more comprehensive to specify here the period of recent fever and recent diarrhea.
Same comments to Vitamin A supplementation and intestinal parasite medication

RECENT is not clear

**Line 259-262**

The variables diarrhea, Fever and Place of residence are not significantly associated to Anemia in multivariate analysis because the confidence interval include “1” (not risk factor)

**Line 287**

It should be Table 3 instead Table 2

**Line 306-308**

This study presents the prevalence in 2005-2010 and 2015 whose results cannot be compared with the 2015 and 2020 objectives.

Instead, the results for 2015 and 2020 should be assessed against the country's objectives for these 2 years.

**Line 311-312**

The country has action plan since 2008. It should mean the country has developed or identified some intervention to address the problem.

If interventions are not implemented in the country to prevent anemia among children, it should be explained by some factors to be highlighted here. If the political commitment does not support strategies, the problem of anemia will always remain a health public health concern in Cambodia.

**Line 314**

Are you referring to univariate or multivariate analysis?

It's important to specify because recent diarrhea is not significantly associated with anemia in multivariate analysis

**Line 319**

The mother's anemia used in this analysis was assessed at what point in the life of the child included in the study?

If it was during the pregnancy of the child evaluated in the survey, the results of this analysis on the link between the child's anemia and that of the mother can be justified, relevant and explained.

On the other hand, if the mother's anemia occurred when the child was already at least 6 months old, I think there could be a bias, otherwise the analysis would have to be taken further, because the link between the child's anemia and the mother's anemia after the child's birth (because the target is children aged 6-59 months) raises questions, unless the mother has a chronic hematological disease.

**Line 333**

In addition, poorly conducted weaning in children aged 6 months and over is a cause of diarrhoeal disease and malnutrition (etc...), a major impact of which could be anemia in children due to nutritional imbalance or insufficient intake of micronutrients, including iron, vitamin B12…..
