# Supplementary material for: Child anemia in Cambodia: A descriptive analysis of temporal and geospatial trends and logistic regression-based examination of factors associated with anemia in children
Source: PLOS Glob Public Health. 2023 Sep 15;3(9):e0002082. doi: 10.1371/journal.pgph.0002082 (PMC10503718; doi:10.1371/journal.pgph.0002082)
Supplement: Attachment — Submitted filename: Response-to-Reviewers.docx [file pgph.0002082.s004.docx]

**Authors’ point-by-point responses to the editor and reviewers**

**PLOS Global Public Health Decision: Revision required [PGPH-D-23-00986] - [EMID:ab165b6dd914812f]**

CC: [sharmarajesh.eco@gmail.com](mailto:sharmarajesh.eco@gmail.com), [rajesh.sharma.dtu@gmail.com](mailto:rajesh.sharma.dtu@gmail.com), [rajesh.sharma@dtu.ac.in](mailto:rajesh.sharma@dtu.ac.in)

PGPH-D-23-00986
Child Anemia in Cambodia: A Descriptive Analysis of Temporal and Geospatial Trends and Logistic Regression-Based Examination of Factors Associated with Anemia in Children Age 6-59 Months

PLOS Global Public Health

Dear Dr. Um,

Thank you for submitting your manuscript to PLOS Global Public Health. After careful consideration, we feel that it has merit but does not fully meet PLOS Global Public Health’s publication criteria as it currently stands. Therefore, we invite you to submit a revised version of the manuscript that addresses the points raised during the review process.

Please submit your revised manuscript by Jul 09 2023 11:59PM. If you will need more time than this to complete your revisions, please reply to this message or contact the journal office at [globalpubhealth@plos.org](mailto:globalpubhealth@plos.org). When you're ready to submit your revision, log on to <https://www.editorialmanager.com/pgph/> and select the 'Submissions Needing Revision' folder to locate your manuscript file.

Please include the following items when submitting your revised manuscript:

- A rebuttal letter that responds to each point raised by the editor and reviewer(s). You should upload this letter as a separate file labeled 'Response to Reviewers'.
- A marked-up copy of your manuscript that highlights changes made to the original version. You should upload this as a separate file labeled 'Revised Manuscript with Track Changes'.
- An unmarked version of your revised paper without tracked changes. You should upload this as a separate file labeled 'Manuscript'.

Guidelines for resubmitting your figure files are available below the reviewer comments at the end of this letter.

We look forward to receiving your revised manuscript.

Kind regards,

Rajesh Sharma, Ph.D.
Academic Editor
PLOS Global Public Health

**Journal Requirements:**

1. Please ensure that the Title in your manuscript and the Title in your online submission form are the same.
   a. We have revised as requested with the title “A Descriptive Analysis of Temporal and Geospatial Trends and Logistic Regression-Based Examination of Factors Associated with Anemia in Children”

2. Please provide separate main figure files in .tif or .eps format only and ensure that all files are under our size limit of 10MB.

For more information about how to convert your figure files please see our guidelines: <https://journals.plos.org/globalpublichealth/s/figures>

a. We have revised accordingly.

3. We noticed that you have two Table 2's in your manuscript. Please update your table numbers and cite them accordingly.

a. We have revised accordingly.

4. We notice that your supplementary tables are included in the manuscript file. Please remove them and upload them with the file type 'Supporting Information'. Please ensure that each Supporting Information file has a legend listed in the manuscript after the references list.

a. We have revised accordingly.

5. Some material included in your submission may be copyrighted. According to PLOS’s copyright policy, authors who use figures or other material (e.g., graphics, clipart, maps) from another author or copyright holder must demonstrate or obtain permission to publish this material under the Creative Commons Attribution 4.0 International (CC BY 4.0) License used by PLOS journals. Please closely review the details of PLOS’s copyright requirements here: PLOS Licenses and Copyright. If you need to request permissions from a copyright holder, you may use PLOS's Copyright Content Permission form.

Please respond directly to this email or email the journal office and provide any known details concerning your material's license terms and permissions required for reuse, even if you have not yet obtained copyright permissions or are unsure of your material's copyright compatibility.

Potential Copyright Issues:

Figure 2: please (a) provide a direct link to the base layer of the map (i.e., the country or region border shape) and ensure this is also included in the figure legend; and (b) provide a link to the terms of use / license information for the base layer image or shapefile. We cannot publish proprietary or copyrighted maps (e.g. Google Maps, Mapquest) and the terms of use for your map base layer must be compatible with our CC-BY 4.0 license.

Note: if you created the map in a software program like R or ArcGIS, please locate and indicate the source of the basemap shapefile onto which data has been plotted.

If your map was obtained from a copyrighted source please amend the figure so that the base map used is from an openly available source. Alternatively, please provide explicit written permission from the copyright holder granting you the right to publish the material under our CC-BY 4.0 license.

Please note that the following CC BY licenses are compatible with PLOS license: CC BY 4.0, CC BY 2.0 and CC BY 3.0, meanwhile such licenses as CC BY-ND 3.0 and others are not compatible due to additional restrictions.

If you are unsure whether you can use a map or not, please do reach out and we will be able to help you. The following websites are good examples of where you can source open access or public domain maps:

* U.S. Geological Survey (USGS) - All maps are in the public domain. ([http://www.usgs.gov](http://www.usgs.gov/)) 
* PlaniGlobe - All maps are published under a Creative Commons license so please cite “PlaniGlobe, [http://www.planiglobe.com](http://www.planiglobe.com/), CC BY 2.0” in the image credit after the caption. (<http://www.planiglobe.com/?lang=enl>) 
* Natural Earth - All maps are public domain. (<http://www.naturalearthdata.com/about/terms-of-use/>)

a. The underlying shapefiles of Cambodia provinces were obtained from the publicly available Spatial Data Repository associated with the DHS [website](https://spatialdata.dhsprogram.com/boundaries/#view=table&countryId=KH). The Spatial Data Repository Database is made available under the [Open Database License](https://opendatacommons.org/licenses/odbl/1.0/). Rights for individual content of the database are licensed under the [Database Contents License](https://opendatacommons.org/licenses/dbcl/1.0/). Additional information provided at [Open Data Commons](https://opendatacommons.org/licenses/odbl/).

6. Please review your reference list to ensure that it is complete and correct. If you have cited papers that have been retracted, please include the rationale for doing so in the manuscript text, or remove these references and replace them with relevant current references. Any changes to the reference list should be mentioned in the rebuttal letter that accompanies your revised manuscript. If you need to cite a retracted article, indicate the article’s retracted status in the References list and also include a citation and full reference for the retraction notice.

Additional Editor Comments (if provided):

[Note: HTML markup is below. Please do not edit.]

Reviewers' comments:

Reviewer's Responses to Questions

**Comments to the Author**

1. Does this manuscript meet PLOS Global Public Health’s [publication criteria](https://journals.plos.org/globalpublichealth/s/criteria-for-publication)? Is the manuscript technically sound, and do the data support the conclusions? The manuscript must describe methodologically and ethically rigorous research with conclusions that are appropriately drawn based on the data presented.

Reviewer #1: Yes

Reviewer #2: Yes

Reviewer #3: Yes

2. Has the statistical analysis been performed appropriately and rigorously?

Reviewer #1: Yes

Reviewer #2: Yes

Reviewer #3: Yes

3. Have the authors made all data underlying the findings in their manuscript fully available (please refer to the Data Availability Statement at the start of the manuscript PDF file)?

The [PLOS Data policy](https://journals.plos.org/globalpublichealth/s/data-availability) requires authors to make all data underlying the findings described in their manuscript fully available without restriction, with rare exception. The data should be provided as part of the manuscript or its supporting information, or deposited to a public repository. For example, in addition to summary statistics, the data points behind means, medians and variance measures should be available. If there are restrictions on publicly sharing data—e.g. participant privacy or use of data from a third party—those must be specified.

Reviewer #1: Yes

Reviewer #2: Yes

Reviewer #3: Yes

4. Is the manuscript presented in an intelligible fashion and written in standard English?

PLOS Global Public Health does not copyedit accepted manuscripts, so the language in submitted articles must be clear, correct, and unambiguous. Any typographical or grammatical errors should be corrected at revision, so please note any specific errors here.

Reviewer #1: Yes

Reviewer #2: Yes

Reviewer #3: Yes

5. Review Comments to the Author

Please use the space provided to explain your answers to the questions above. You may also include additional comments for the author, including concerns about dual publication, research ethics, or publication ethics. (Please upload your review as an attachment if it exceeds 20,000 characters)

Reviewer #1: Title needs modification. It needs to be shorter, easily understandable and coherent to the objective of the study.

a. We have revised the title so that is concise but also comprehensive in its description of our study.

Don't forget to justify why you haven't used the data if there is any Demographic and Health Survey undertaken after 2014 in the country.

a. Thank you for this guidance. We have addressed this as a limitation within the discussion section of the manuscript. The most recent round of the Cambodia Demographic and Health Survey (CDHS) was for 2022. Unfortunately, data on Hemoglobin/Anemia were not collected from children or women. We have acknowledged that this may no longer reflect present prevalence of anemia among children in Cambodia. However, we have also cited WHO estimates that suggest the patterns that we’ve described have continued on at least through 2019. We also believe there is an important contribution for conveying thee temporal and geospatial trends of anemia and associated factors among children over the 15-year period for which data are available as this can serve as a baseline for future comparisons and research.

There are scholars and organizations which classify those with mild anemia as 'not anemic'. Consider if this apply in your case.

a. We used the World Health Organization (WHO) classification criteria, which stipulates that children aged 6-59 months are considered anemic if their hemoglobin level is below 11.0 g/dL (<https://www.who.int/data/nutrition/nlis/info/anaemia>). Accordingly, children with mild, moderate, and severe were all classified as anemic.

Better to mention if there was any data quality control measures undertaken especially Hemoglobin adjustments.

a. We have addressed this as suggested and highlighted in the revised manuscript about hemoglobin level measurement in the Subheading “**Data Source and Procedures”.** Hemoglobin level in capillary blood was assessed using HemoCue 201+ testing technique, adjusted for altitude and cigarette smoking. The detailed process has been shown on the website (<https://dhsprogram.com/topics/Anemia.cfm>).

Recommendations has to be specific per the findings. If there are interventions being underway scaling it up and strengthening it also has to be considered.

a) We have expanded our discussion section to include information related to interventions and policies that have been implemented to address this public health challenge. However, the persistence of anemia among children as indicated by WHO estimates suggest that more still needs to be done to address geospatial disparities.

Reviewer #2:

Comments
1.Line 122-124 how the researchers arrived at the 10,434 is not clear. Further explanation on this will be helpful.

a. Thank you for this recommendation. We have addressed this as suggested and highlighted in the revised manuscript about the final sample size selection process (**see Fig 1**.) in the Subheading “**Data Source and Procedures.”**

2.The summation of the frequencies (computation) in Table 1 under the Characteristics of Place of birth under section Home does not add up. Authors are encouraged to check these figures correctly.

a. We reviewed and have revised Table 1 in the manuscript.

- Furthermore, the authors failed to perform dietary assessment on the mothers and again whether the babies where exclusively fed.

a. Yes, we have mentioned in our discussion of the limitations in our study.

4. In my candid opinion based on the multiple limitation I suggest the title is modified slightly to represent the findings given that data was primarily a self-reported type.

a. We have revised the title so that it is concise but also comprehensive in its description of our study. We have also endeavored to represent the study transparently in describing its limitations in the discussion section.

5.The pre-maternal weights of the mothers could have been captured since these are normally in their prenatal books/records.

a. We agree that this would be interesting to include in our analysis, but we are unfamiliar with the availability of pre-maternal weights for women in the DHS survey. We searched the supporting documentation for the data and did not find these types of data available. We were only able to find variables for maternal weight at the time of the survey.

6.While the findings are interesting, I am not sure the findings represent the current situation given that the data were collected almost a decade ago. Will it be relevant today and what is the current situation of children anemia in Cambodia today?

a. We appreciate this feedback and have now cited estimates from the WHO that indicate that prevalence of anemia declined only slightly between 2015 and 2019 and was still quite high going into 2019/2020. Unfortunately, the most recent Demographic and Health Survey for Cambodia (2022) did not include information on hemoglobin measurements for children, so we are unable to assess empirically the extent to which our findings are a reflection of current conditions. Rather, we have endeavored to do the best we can with the data available using CDHS from 2005, 2010, and 2014 and then citing external sources to provide a more recent context. We have also acknowledged this as a data limitation in our discussion section.

Reviewer #3:

Line 69

Which period? Please specify the period and reference

a. We revised accordingly.

Line 136-140

Can SINGLE, WIDOWED and SEPARATED women be considered married?
In reality their social status is "NOT MARRIED" as they are not married.

a. We revised accordingly.

Same comments with OCCUPATION. It would be good to make the following groups:

1. NOT WORKING (NO INCOME)
2. WORKING (others who work and earn money for family needs, so they have INCOME)

a.	We revised and have not referenced this as employment rather than occupation.

Line 145-146

Place of birth should be “HOSPITAL" and "HOME" as being born in a public or private hospital is the same government objective (birth assisted by qualified personnel).

a.	We revised accordingly.

Indicators monitor are “Delivery assisted by qualified health worker” and “Delivery unassisted by qualified worker”

a.	We revised accordingly.

Line 148-150

It would be more comprehensive to specify here the period of recent fever and recent diarrhea.
Same comments to Vitamin A supplementation and intestinal parasite medication
RECENT is not clear

a.	We revised accordingly.

Line 259-262

The variables diarrhea, Fever and Place of residence are not significantly associated to Anemia in multivariate analysis because the confidence interval include “1” (not risk factor)

a.	We revised accordingly.

Line 287

It should be Table 3 instead Table 2

a.	We revised accordingly.

Line 306-308

This study presents the prevalence in 2005-2010 and 2015 whose results cannot be compared with the 2015 and 2020 objectives. Instead, the results for 2015 and 2020 should be assessed against the country's objectives for these 2 years.

a.	We revised the manuscript toward a more direct comparison of comparable time periods.

Line 311-312

The country has action plan since 2008. It should mean the country has developed or identified some intervention to address the problem. If interventions are not implemented in the country to prevent anemia among children, it should be explained by some factors to be highlighted here. If the political commitment does not support strategies, the problem of anemia will always remain a health public health concern in Cambodia.

a. Thank you, we have revised the manuscript to include additional details concerning policies and interventions in our discussion section.

Line 314

Are you referring to univariate or multivariate analysis? It's important to specify because recent diarrhea is not significantly associated with anemia in multivariate analysis

a.	We have corrected this, thank you.

Line 319

The mother's anemia used in this analysis was assessed at what point in the life of the child included in the study? If it was during the pregnancy of the child evaluated in the survey, the results of this analysis on the link between the child's anemia and that of the mother can be justified, relevant and explained. On the other hand, if the mother's anemia occurred when the child was already at least 6 months old, I think there could be a bias, otherwise the analysis would have to be taken further, because the link between the child's anemia and the mother's anemia after the child's birth (because the target is children aged 6-59 months) raises questions, unless the mother has a chronic hematological disease.

a. Maternal anemic status was assessed at the time of the study. Given the timing of the measurement, we have noted in our discussion that the mechanisms at play are likely in the realm of social determinants with mothers and children sharing socioeconomic status and household access to food. However, other plausible explanations are also discussed, though we cannot empirically test for these pathways given the temporality of when anemia status was measured.

Line 333

In addition, poorly conducted weaning in children aged 6 months and over is a cause of diarrhoeal disease and malnutrition (etc...), a major impact of which could be anemia in children due to nutritional imbalance or insufficient intake of micronutrients, including iron, vitamin B12…..

a. We have endeavored to address this in our discussion section.

6. PLOS authors have the option to publish the peer review history of their article ([what does this mean?](https://journals.plos.org/globalpublichealth/s/editorial-and-peer-review-process#loc-peer-review-history)). If published, this will include your full peer review and any attached files.

**Do you want your identity to be public for this peer review?** If you choose “no”, your identity will remain anonymous but your review may still be made public.

For information about this choice, including consent withdrawal, please see our [Privacy Policy](https://www.plos.org/privacy-policy).

Reviewer #1: **Yes:** Shalama Lekasa Nagari

Reviewer #2: No

Reviewer #3: **Yes:** Kouadio Kobahounde, Epidemiologist
